# Supplementary material for: Identification of photoperiod-induced specific miRNAs in the adrenal glands of Sunite sheep (Ovis aries)
Source: Front Vet Sci. 2022 Jul 22;9:888207. doi: 10.3389/fvets.2022.888207 (PMC9354845; doi:10.3389/fvets.2022.888207)
Supplement: Supplementary file 1 [file Data_Sheet_1.ZIP › Supplementary file/miRDeep2 file.zip!/miRDeep2 file/Know_Structure/SPLP42A2/oar-mir-543.pdf]

miRBase precursor : oar-mir-543
Total read count : 15986
oar-miR-543-5p read count : 7
oar-miR-543-3p read count : 15938
remaining reads : 41

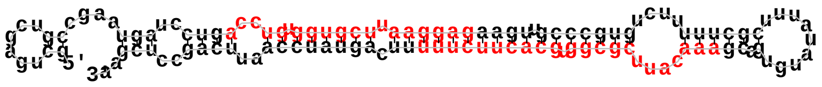

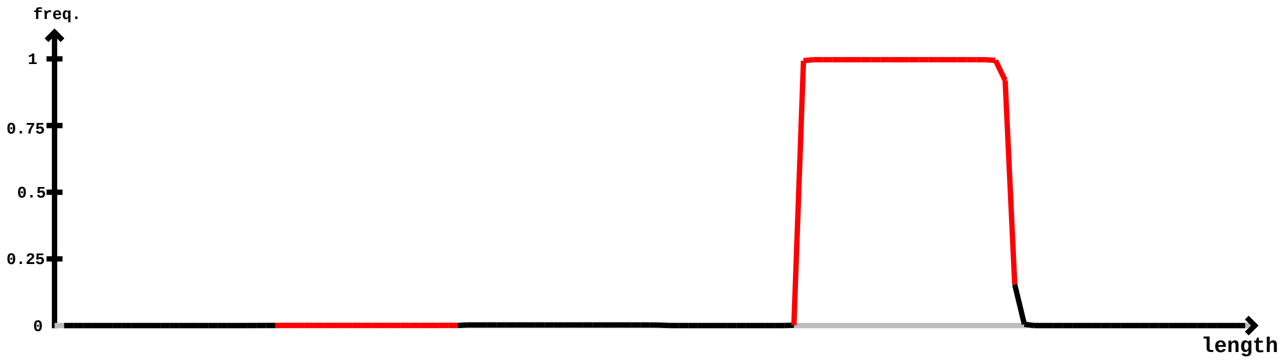


```
                                                                                oar-miR-543-3p
                            oar-miR-543-5p
5'- gcugagcugccgaaugauccugaccuguggugcuuaaggagaaguugcccgugucuuuuucgcuuuauaugugacgaaacauucgcggugcacuucuuuuucaguauccaauucagccucgaa  -3'  exp
    ((......)).....((..((((..((.((((((.((((((((((.(((((((....((((((.........).)))))....))))).)))))))))))).))))))))..))))..))...       reads  mm
    ..................ccugaccuguggugcuuaagga...................................................................................       1      0
    ...................cugaccuguggugcuuaag.....................................................................................       1      0
    ...................cugaccuguggugcuuaagga...................................................................................       1      0
    ....................ugaccuguggugcuuaagga...................................................................................       2      0
    .....................gaccuguggugcuuaagga...................................................................................       2      0
    ......................accuguggugcuuaagga...................................................................................       3      0
    .......................................agaaguugcccgugucuuuu................................................................       1      0
    .......................................agaaguugcccgugucuuuuuc..............................................................       3      0
    .......................................agaaguugcccgugucuuuuucgc............................................................       1      0
    ........................................gaaguugcccgugucuuuuucg.............................................................       1      0
    .........................................aaguugcccgugucuuuuuc..............................................................       5      0
    .........................................aaguugcccgugucuuuuucg.............................................................       25     0
    .........................................aaguugcccgugucuuuuucgcu...........................................................       2      0
    ..........................................................................cgaaacauucgcggugcacuuc...........................       1      0
    ..........................................................................cgaaacauucgcggugcacuucu..........................       1      0
    ...........................................................................gaaacauucgcggugcacuucu..........................       17     0
    ...........................................................................gaaacauucgcggugcacuucuu.........................       3      0
    ............................................................................aaacauucgcggugcacu.............................       1      0
    ............................................................................aaacauucgcggugcacuu............................       3      0
    ............................................................................aaacauucgcggugcacuuc...........................       38     0
    ............................................................................aaacauucgcggugcacuucu..........................       1173   0
    ............................................................................aaacauucgcggugcacuucuu.........................       12192  0
    ............................................................................aaacauucgcggugcacuucuuu........................       2370   0
    ............................................................................aaacauucgcggugcacuucuuuu.......................       77     0
    ............................................................................aaacauucgcggugcacuucuuuuu......................       1      0
    .............................................................................aacauucgcggugcacuucu..........................       2      0
    .............................................................................aacauucgcggugcacuucuu.........................       43     0
    .............................................................................aacauucgcggugcacuucuuu........................       13     0
    .............................................................................aacauucgcggugcacuucuuuu.......................       1      0
    ...............................................................................cauucgcggugcacuucuu.........................       1      0
    .................................................................................uucgcggugcacuucuu.........................       1      0
```
